# Synthesis and Characterization of Ru-MOFs on Microelectrode for Trace Mercury Detection †

**DOI:** 10.3390/s20226686

**Published:** 2020-11-23

**Authors:** Chenyu Xiong, Yuhao Xu, Chao Bian, Ri Wang, Yong Xie, Mingjie Han, Shanhong Xia

**Affiliations:** 1 State Key Laboratory of Transducer Technology, Aerospace Information Research Institute, Chinese Academy of Sciences, Beijing 100190, China; xiongchenyu16@mails.ucas.ac.cn (C.X.); xuyuhao15@mails.ucas.ac.cn (Y.X.); cbian@mails.ie.ac.cn (C.B.); wangri17@mails.ucas.ac.cn (R.W.); xieyong16@mails.ucas.ac.cn (Y.X.); mjhan1993@outlook.com (M.H.); 2 School of Electronic, Electrical and Communication Engineering, University of Chinese Academy of Sciences, Beijing 100049, China

**Keywords:** mercury ions, metal-organic frameworks, electrochemical micro-sensor, cathodic synthesis

## Abstract

Mercury ions (Hg${}^{2+}$) pollution in the water environment can cause serious harm to human health. Trace Hg${}^{2+}$ detection is of vital importance for environmental monitoring. Herein, we report a novel design of Ru-MOFs modified gold microelectrode for Hg${}^{2+}$ determination. Ru-MOFs are synthesized directly by the cathodic method on gold microelectrode, with the covered area accurately controlled. Cathodic synthesized Ru-MOFs show good conductivity and are suitable to be used as the electrode surface material directly. The synergy of the pre-deposition process and the adsorption process of Ru-MOFs can effectively improves the performance of the sensor. The results show good linearity (R${}^{2}$ = 0.996) from 0.1 ppb to 5 ppb, with a high sensitivity of 0.583 μA ppb${}^{-1}$ mm${}^{-2}$. The limit of detection is found to be 0.08 ppb and the test process is within 6 min. Most importantly, the senor has a good anti-interference ability and the recoveries are satisfactory. This miniature electrochemical sensor has the potential for on-site detection of trace mercury in the field.

## 1. Introduction

Hg${}^{2+}$ is biologically toxic [1] and once digested in the body can cause neurological and cognitive disorders [2]. Even though the concentration of mercury is at a very low level in natural water, it would be a potential threat to the ecosystem and human health due to its high enrichment factor (up to 10${}^{6}$) [3]. Strict legislation limits of its concentration are imposed in many countries, for example, the United States Environmental Protection Agency (US-EPA) regulation recommends that the Hg${}^{2+}$ in potable water should be under 2 ppb. It is necessary to develop an accurate and onsite method to detect or monitor trace Hg${}^{2+}$ pollution in the water environment. Cold vapor atomic fluorescence spectrometry(CV-AFS) [4], cold vapor atomic absorption spectrometry (CV-AAS) [5,6] and inductively coupled plasma mass spectrometry (ICP-MS) [7,8] are the most popular methods that detect trace mercury in the ppt level, but these methods require large, expensive instrumentation and complex operation, which are not the best choice for trace Hg${}^{2+}$ on-site detection in the field. Recently, several methods such as fluorimetry [9,10], colorimetry [11,12], raman spectrometry [13,14,15] and localized surface plasmon resonance [16] were used for Hg${}^{2+}$ detection. However, most of these methods have low sensitivity, labelling requirements and high-cost instruments requirements. Contrariwise, the electrochemical sensor has its advantages in economy, portability and easy integration. Among electrochemical detection methods, anodic stripping voltammetry has been widely used for trace detection because of its convenience, portability, high sensitivity, low limit of detection (LOD) and real-time monitoring [17,18,19]. However, there is still a challenge on how to enhance the specific active sites and improve the electrochemical activity, which severely restricts the Hg${}^{2+}$ sensing performance and limits its utilization in trace detection of Hg${}^{2+}$ in the natural water system.

Metal-organic frameworks (MOFs), a class of porous material, has attracted increasing research attention because of its high surface area, porosity, large pore volume, tunable structures, excellent adsorption, tailorable pore sizes and functionality. MOFs are served as an attractive material for gas and energy storage [20,21,22], adsorption [23], separation [24,25,26], sensing [27,28,29], drug delivery [30] and catalysis [31,32,33]. In recent studies, MOFs have been used for the capture or detection of some metal ions. For example, the bismuth-based metal-organic framework can selectively capture toxic SeO${}_{3}^{2-}$ [34]. MOFs derived iron oxide-based smart plasmonic Ag/Au hollow and porous nanoshells electrodes can be used for ultra-sensitive detection of trace arsenic [18]. Graphene aerogel–MOFs have been reported for the simultaneous detection of multiple ions [35]. However, the electronic conductivity of MOFs is poor. Therefore, improving the conductivity has been an important research focus in recent years. For this respect, integrating MOFs and conductive materials, such as metal nanoparticles, graphene-based materials and carbon materials, into a single nanostructure is a major effective method to improve MOFs conductivity [32,36,37,38]. However, the materials synthesized by this method need to be spin-coated on the surface of the electrode, so the approach is difficult to control the precision of the covered area. As an alternative, the cathode synthesis method can directly deposit MOFs on the electrode surface. Compared with traditional hydrothermal synthesis, the cathode method has short synthesis time and mild conditions [39,40,41,42,43], and can directly synthesize MOFs on the electrode. For the cathode synthesized MOFs, its lattice structure is prone to defect and it contains unsaturated metal centers, which improves the conductivity of the material. To be the best of our knowledge, the electrochemical sensing application of MOFs modified microelectrode has not been reported yet.

Herein, for the first time, we present a strategy to directly synthesize Ru-MOFs on gold microelectrodes. The Ru-MOFs modified gold microelectrode (Ru-MOFs@Au microelectrode) was used as an electrochemical sensor for the trace mercury sensing. In our study, the gold disc microelectrode was prepared by Micro Electro-Mechanical System (MEMS) technique. Then the Ru-based metal-organic frameworks were directly synthesized on the gold microelectrode surface by electrochemical cathodic synthesis method. In the process of cathodic synthesis, Ru-MOFs was thus synthesized in dimethylformamide. Tetrabutylammonium hexafluorophosphate (TBAPPF${}_{6}$) was used as the electrolyte. The gold microelectrode was utilized as the cathode. Ruthenium chloride was selected as the metal source. The organic ligands of 1,3,5-benzenetetracarboxylic acid (H${}_{3}$BTC) were used as organic ligands. Ru-MOFs show a good electrochemical activity and improve the current response of microelectrode to Hg${}^{2+}$. The Ru-MOFs modified MEMS-based gold microelectrode shows a high electrocatalytic activity and has a high sensitivity (0.583 μA ppb${}^{-1}$ mm${}^{-2}$) to Hg${}^{2+}$. We realized the ultra-sensitive detection of mercury and effectively reduced the detection limit. The electrochemical microsensor has the potential for rapid and on-site detection of trace Hg${}^{2+}$.

## 2. Exeprimental Section

### 2.1. Reagents

AZ1500 was purchased from AZ Electronic Materials Company. Su-8 was purchased from MicroChem Company. Ruthenium chloride (RuCl3) was purchased from Sigma (St. Louis, MO, USA). 1,3,5 benzenetricarboxylic (98%, H${}_{3}$BTC), tetrabutylammonium hexafluorophosphate (98%, TBAPPF${}_{6}$) and sodium hydroxide (NaOH, 99.9%) were obtained from Aladdin Chemistry Co., Ltd. (Shanghai, China). N,N-dimethylformamide (99.5%, DMF) was obtained from Beijing Chemical Works (Beijing, China). Mercury standard stock solution (100 ppm Hg${}^{2+}$ with 3% nitric acid) was purchased from the China National Research Centre for Certified Reference Material. All deionized water was prepared daily by the Millipore DQ3UV water purification system (Millipore Company, Darmstadt, Germany) at room temperature.

### 2.2. Apparatus

In this work, the synthesis of Ru-MOFs and all the electrochemical experiments were carried out on an electrochemical workstation (Gamry Reference 600, Gamry Instruments, Warminster, PA, USA) by the three-electrode system. The gold disc microelectrode (diameter: 1mm) fabricated by MEMS technology was used as a working electrode. A platinum electrode (diameter: 2 mm) and Ag/AgCl (3M KCl, aq) electrode were used as counter and reference electrode respectively. The mercury concentrations in tap water were examined with atomic fluorescence spectrometry by the PONY Testing International Group (PONY Company, Beijing, China). Scanning electron microscopy (SEM) images of the synthesis Ru-MOFs layer was performed by using the A-4800 field emission electron microscope (FESEM) produced by Hitachi (Tokyo, Japan).Ultra-high vacuum magnetron sputtering equipment (JGD560B3) was produced by Sky Technology development (Liaoning, China). Dicing machine (ZSH428) was produced by Shenyang Acadamy of Instrumentation Science CO., LTD (Liaoning, China). Unless otherwise specified, all experiments are carried out at room temperature (25 celsius).

### 2.3. Fabrication of Gold Microelectrodes

**Figure 1:**
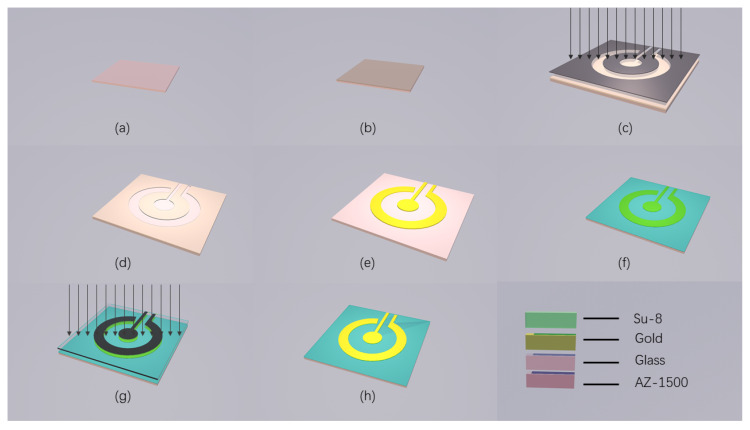
The fabrication processes of the gold micro-electrode. (**a**,**b**) Step to prepare glass slide and glue. (**c**–**e**) Step to pattern metal layer and deposit gold layer. (**f**–**h**) Step to prepare the Su-8 layer.

The gold disc microelectrode (Au electrode) was prepared by MEMS technology and the whole process was completed in the cleanroom. As illustrated in Figure 1, the fabrication process involved lithography on the glass substrate, followed by sputtering and stripping to obtain the pattern of gold disc microelectrode (GDM). Firstly, positive photoresist AZ1500 was spun on the metal layer at 1000 rpm for 60 s (Figure 1b). Then, the sample was cured on a hot plate at 90 degrees Celsius and was strictly controlled at 90 degrees Celsius for 5 min. After lithography (Figure 1c), the photoresist was developed in 0.6% NaOH, and hard-baked at 60 degrees Celsius (Figure 1d). Oxygenating the sample for 30 s to remove residual glue, and then sputter it with 300 Å Ta and 2000 Å Au (Figure 1e). After that, immersing the sample in ethyl ketone for 5 h to remove the photoresist. At this time, we needed to make sure that the working electrode was disconnected from the counter electrode. After the oxygen plasma treatment, the remaining photoresist was removed. Then, the negative photoresist SU-8 was spin-coated on the surface of the sample at a rotation speed of 3000 rpm for 60 s (Figure 1f), and the sample was heated on a hot plate at 90 degrees Celsius for 20 min and then cooled naturally. After photolithography (Figure 1g), the sample was developed in isopropanol (Figure 1h). After dicing, patching, oxygenation, pressure welding and packaging, the disk microelectrode was obtained. Finally, after oxygenation, the electrode surface was cleaned.

### 2.4. Synthesis of the Ru-MOFs

Firstly, the fabricated microelectrode by the Micro Electro-Mechanical System (MEMS) was cleaned by the ultrasonic method in deionized water, then the microelectrode was activated in dilute sulfuric acid using cyclic voltammetry (CV) with a voltage range from 0 V to 1.5 V for at least 5 times. Secondly, 0.01 M 1,3,5 benzenetricarboxylic (H${}_{3}$BTC, 42.028 mg) and 0.01 M tetrabutylammonium hexafluorophosphate (TBAPPF${}_{6}$, 77.486 mg) were added into 16 ml N, N-dimethylformamide (DMF). Then 1.4 mL deionized water, 0.6 mL 1M nitric acid (HNO${}_{3}$) and 2 mL 0.1 M ruthenium chloride (RuCl${}_{3}$) were add into the mixture solution. The mixture was ultrasonic stirred for 10 min and stood still for a while. Finally, Ru-MOFs films were prepared on the surface of a working microelectrode in the mixture solution by the cathodic synthesis within fifteen minutes at room temperature. Chronoamperometry was used to synthesize Ru-MOFs at −1.3 V (vs ref) for 15 min at room temperature. DMF was used to remove impurities from the electrode surface, then deionized water was used to remove DMF. This process was repeated 2–3 times. After these steps, the electrode was heated to 50 degrees Celsius for 30 min in an oven. For the process of synthesis, DMF and deionized water were used as co-solvent, RuCl${}_{3}$ was served as a metal source. The organic linker was H${}_{3}$BTC, TBAPPF${}_{6}$ was served as the electrolyte and nitric acid was used as the reaction medium.

### 2.5. Electrochemical Measurements

Different concentrations of mercury solution were obtained by stepwise diluting mercury standard solution with a concentration of 100 ppb. Next, 1 mL 1 M hydrochloric acid (HCl) was added into the 9 mL sample. Then, a 10 mL test sample was detected using three-electrode system. the Ru-MOFs@Au microelectrode was served as the working electrode (WE), platinum electrode and Ag/AgCl (3 M KCl, aq) electrode were used as counter and reference electrode respectively. The measuring process mainly included three parts. First, the potential of Ru-MOFs@Au microelectrode was held at 0.8 V (vs. ref) for 60 s to clean the electrode surface. Second, the differential plus string voltammetry (DPSV) was used for the detection of trace mercury. The potential of WE was maintained at −0.8 V for 240 s, the Hg${}^{2+}$ would be enriching around WE surface due to adsorption of multi-vacancy structure and attraction of negative potential, and the Hg${}^{2+}$ was reduced to Hg${}^{0}$, which made Hg${}^{0}$ continuously enriched on the WE surface. Then the potential scanned from low to high with a voltage range from 0 V to 0.8 V, Hg${}^{0}$ was stripped from the electrode surface and oxidized to Hg${}^{2+}$. There would be an oxidation current peak and the stripping peak was related to the Hg${}^{2+}$ concentration. After the last test was completed, the WE was held at a high potential again to clean the surface of the electrode, which ensured that the reduced Hg${}^{0}$ was completely oxidized back into the test solution.

## 3. Results and Discussion

### 3.1. The Process of Cathodic MOFs Deposition

**Figure 2:**
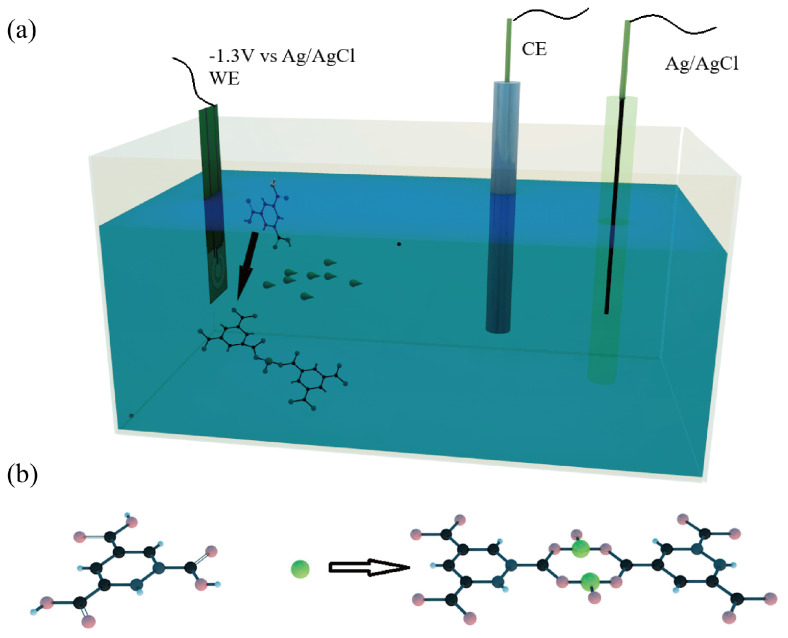
(**a**) Synthesis of Ru-MOFs by electrochemical cathode synthesis. (**b**) representative modulated synthesis

The well-attached films of Ru-MOFs were rapidly synthesized within fifteen minutes at room temperature by the cathodic electrochemical method. The method directly deposited Ru-MOFs on the microelectrode surface, which effectively controlled the covered area on the microelectrode. The synthesis time of this electrochemical synthesis method is very important because too short a time will result in an incomplete dense crystal layer, while a longer synthesis time will cause the MOFs to be too thick and fall off. We found that when the working electrode was kept at −1.3 V for 15 min to cathodic synthesis, the Ru-MOFs films were completely covered the electrode surface and did not appear to be too thick or fall off. The mechanism of cathodic synthesis has already been studied and described in recent researchs [44,45,46]. In this work, The process of synthesis is shown in Figure 2a, a large amount of ruthenium ions was attracted by negative potential then gathered around the cathode, which caused the concentration of ruthenium ions near the cathode to increase. The reduction of nitrates (1) caused a shift in pH near the electrode surface. The deprotonation of organic ligand (3) is triggered due to the shift in pH (1) (2), and the hydrogen evolution (2) (4) at the cathode moved the equilibrium and promoted the deprotonation. Then the deprotonated linkers (BTC${}^{3-}$) and high concentration of ruthenium ions near the cathode triggered self-assembly (5) which led to the formation of Ru-MOFs nuclei on the electrode surface, followed by the growth of islands and intergrowth. Finally, the Ru-MOFs films were deposited on the gold disc electrode. The reaction equations are as follows.

(1) $$NO_{3}^{-}+2e^{-}+2H^{+}⇌NO_{2}^{-}+H_{2}O$$

(2) $$NO_{2}^{-}+4e^{-}+3H_{2}O+2H^{+}⇌NO_{3}^{-}+2OH^{-}+2H_{2}$$

(3) $$3OH^{-}+H_{3}BTC⇌3BTC^{3-}+3H_{2}O$$

(4) $$2H_{2}O+2e^{-}⇌2OH^{-}+H_{2}$$

(5) $$Ru^{3+}+BTC^{3-}⇌Ru-MOFs$$

### 3.2. Performance of Ru-MOFs

**Figure 3:**
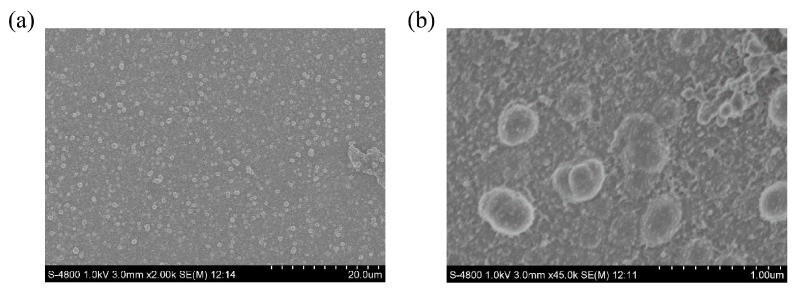
SEM images of Ru-MOFs with magnification of (**a**) 2K and (**b**) 45K.

**Figure 4:**
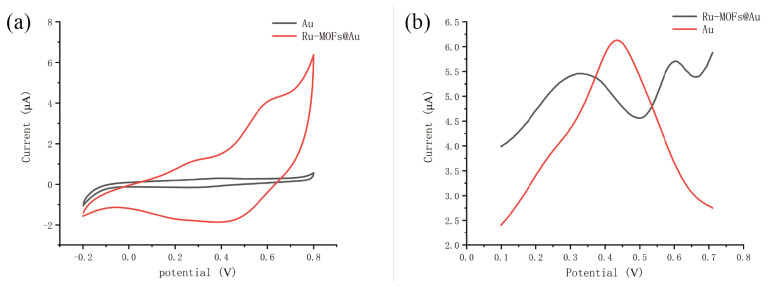
(**a**) CV scan from −0.2 V–0.8 V in 0.1 M KCl. (**b**) DPSV in 5 ppb Hg${}^{2+}$ solution, the deposition condition was 120 s at −0.8 V.

The SEM images of Ru-MOFs were shown in Figure 3, in which the Ru-MOFs can be seen clearly. In order to test the electrochemical performance of the synthesized MOFs, the electrochemical activity of Au and Ru-MOFs@Au microelectrode were checked in 0.1M KCl by cyclic voltammetry. The potential of the electrode was scanned from −0.2 V to 0.8 V in 0.1 M KCl. As shown in Figure 4a, compared with the gold microelectrode, the current response of Ru-MOFs@Au electrode was larger than the gold microelectrode. It indicated that cathodic synthesized Ru-MOFs have good conductivity and can be directly used as the electrode material, and synthesis of Ru-MOFs on the surface of the gold electrode significantly improved electrochemical activity of the electrode surface. Note that, two pairs of oxidation peaks were observed in 0.1 M KCl, which indicated there might be exposed ruthenium active centers in Ru-MOFs. The unsaturated metal center could effectively adsorb metal ions and provide chemical reaction sites.

As shown in Figure 4, Ru-MOFs show a significant electrochemical activity and the current response of microelectrode to Hg${}^{2+}$. Figure 4a shows the cyclic voltammetry characteristic curve of the gold electrode and the electrode modified with Ru-MOFs in 0.1 M KCl. The scan ranges from −0.2 V to 0.6 V. This indicates that the modified Ru-MOFs effectively improve the electrocatalytic activity of the electrode. Note that, a peaks were observed in 0.1 M KCl at 0.55 V, the wave result from the oxidation process of exposed ruthenium active inside Ru-MOFs, probably in reaction forms of Ru(II)-e${}^{-}$⇌ Ru(III) for the wave at 0.55 V. Figure 4b shows the current response curve of two electrodes (Au microelectrode and Ru-MOFs@Au electrode) to mercury ions. After the electrode is modified with Ru-MOFs, the response of the electrode to Hg${}^{2+}$ increases significantly. It indicates that Ru-MOFs improve the sensor’s sensitivity to Hg${}^{2+}$, which is mainly attributed to the increase in electrocatalytic activity and the outstanding adsorption capacity of Ru-MOFs.

### 3.3. Mercury Determination with the Ru-MOFs Modified Microelectrode

**Figure 5:**
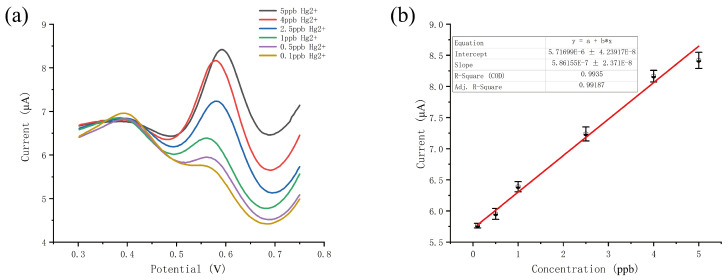
(**a**) current response of different Hg${}^{2+}$ concentrations; (**b**) liner response curve to Hg${}^{2+}$.

We found that the Ru-MOFs@Au electrode shows a great response to trace Hg${}^{2+}$, so we use the electrode to detect Hg${}^{2+}$ in different concentration. The response curve is shown in Figure 5a and the linear curve is shown in Figure 5b. The result indicated that Ru-MOFs modified microelectrode can be used for the detection of trace Hg${}^{2+}$ in aqueous solution. As shown in Figure 5a,b, as the increase of Hg${}^{2+}$, the oxidation current increased. The linear response range was from 0.1 ppb to 5 ppb with a correlation coefficient of 0.996. The LOD is 0.08 ppb and the sensitivity is 0.583 μA ppb${}^{-1}$ mm${}^{-2}$. This small size sensor can detect trace mercury rapidly as the whole test process costs time within 6 min. These results indicated that the sensor has the potential for trace Hg${}^{2+}$ detection in the field.

### 3.4. Anti-Interference Test

**Figure 6:**
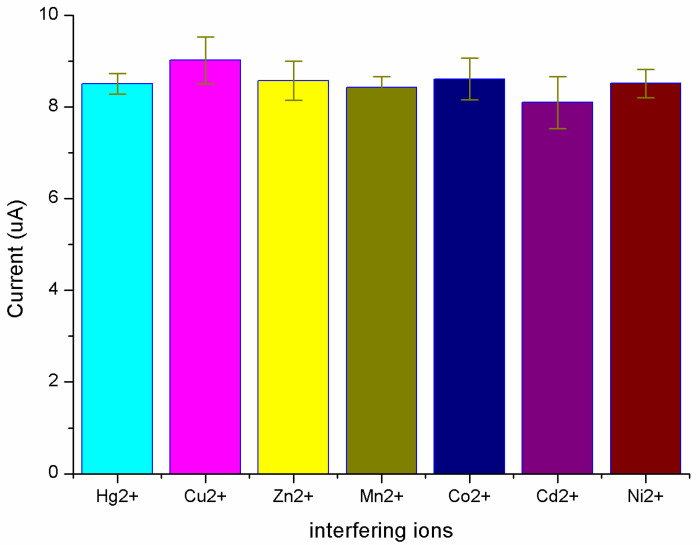
Current peak in the presence of 50 ppb marked metal ions followed by addition of 5 ppb Hg${}^{2+}$ with the blank solution subtracted.

The effect of coexistence interference ions for mercury detection was studied. In these experiments, solutions of 5 ppb Hg${}^{2+}$ spiked with 50 ppb various metal interference ions were prepared to demonstrate the anti-interference of the Ru-MOFs@Au microelectrode. Firstly, 5 ppb Hg${}^{2+}$ test sample with 0.1 M HCl and 5 ppm interference aqueous solution were prepared. Then 20 μL interference ions solution was added into 2 mL prepared Hg${}^{2+}$ solution. In the test sample, the concentration of interference ions was 10 times as much as the concentration of Hg${}^{2+}$. Cu${}^{2+}$, Zn${}^{2+}$, Mn${}^{2+}$, Co${}^{2+}$, Cd${}^{2+}$ and Ni${}^{2+}$ were chosen as the interference ions since they are divalent cations, which have similar electrochemical properties to Hg${}^{2+}$. Figure 6 shows the current response to the presence of mercury and other interference ions solutions, with the current peak of blank solution subtracted. The current peak almost remained unchanged with the addition 10 times concentration of other interference ions, including Cu${}^{2+}$, Zn${}^{2+}$, Mn${}^{2+}$, Co${}^{2+}$, Cd${}^{2+}$ and Ni${}^{2+}$. The Ru-MOFs@Au microelectrode displayed strong anti-interference ability.

### 3.5. Application to the Water Sample

In order to investigate the potential of the sensor for trace mercury detection in water sample, top water collected in our lab were detected. First, the concentration of Hg${}^{2+}$ in the initial tap water sample was certified using ICP-MS.The basic characterization of tap water was measured by commercial instrumentations, the pH, dissolved oxygen (DO), temperature and conductivity are 7.84, 4.36 mg/L, 25 Celsius and 293 μS respectively. Next, the sample was firstly spiked with 0.5 ppb, 2 ppb and 4 ppb Hg${}^{2+}$ from the standard stock solution. Then the concentration of Hg${}^{2+}$ in mixture water sample was analyzed with our method. The results are shown in Table 1, the recoveries of Hg${}^{2+}$ ranged from 93.5% to 108.5%, the sensor has the potential for the trace Hg${}^{2+}$ detection in water samples with complex matrices.

### 3.6. Comparision with Other Methods

In our work, the microelectrode detected trace mercury by anodic stripping analysis, so the performance of the sensor was compared with several reports of trace mercury determination using anodic stripping voltammetry. The LOD in our work is better than those of most methods in Table 2. As the porosity material, Ru-MOFs show good adsorption capacity and improve the electrocatalytic activity in our work, which efficiently increases the sensitivity to Hg${}^{2+}$ and reduces the LOD of the sensor.

## 4. Conclusions

In summary, an electrochemistry based on the Ru-MOFs@Au microelectrode was proposed and experimentally for the detection of trace mercury ions. The gold microelectrode was fabricated by the MEMS technology, then Ru-MOFs were synthesized on a MEMS-based gold microelectrode by cathodic method, with the covered area of microelectrode accurately controlled. Electrochemical performance analysis using CV showed that cathodic synthesized Ru-MOFs have good conductivity and are suitable to be used as the sensing material. Further experiments demonstrated that the Ru-MOFs modified gold electrode can be used for trace mercury ions detection with high sensitivity, strong anti-interference capability and good recovery. All the results suggested that the sensor has the potential for the determination of trace mercury.

It must be noticed that the performance of such sensors may be influenced by extraneous parameters as temperature. Therefore, in such case a background single (without Hg${}^{2+}$) should be detection to compensate for these parameters. In the future, we plan to further study the sensor’s performance in natural water and the strategy of temperature compensation, which improve sensor’s general applicability in natural water. 

## Figures and Tables

**Table 1:** Analytical result of Hg${}^{2+}$ by the DPSV method in tap water.

Sample	Found (ppb)	Added (ppb)	Found (ppb)	Recovery
Tap water 1	N.D	0.5	0.53 ± 0.03	106%
Tap water 2	N.D	2	2.17 ± 0.12	108.5%
Tap water 3	N.D	4	3.74 ± 0.28	93.5%

**Table 2:** Comparison of analytical performances with other anodic stripping methods for the determination of trace mercury in aqueous sample.

Electrode	Method	Linear Detection Range	Detection Limit	Reference
BieAuNPs@CPE	SWASV	2.6 nM –997 nM	1.5 nM	[47]
GR-CD@PPy@SPCE	DPASV	1 nM–57.557 μM	0.47 nM	[48]
SePs-AuNPs@CPE	DPASV	69.8 nM–17.4 μM	5.1 nM	[49]
Ru-MOFs@AuME	DPSV	0.495 nM–24.75 nM	0.39 nM	This work
